# Impact of today's media on university student's body image in Pakistan: a conservative, developing country's perspective

**DOI:** 10.1186/1471-2458-11-379

**Published:** 2011-05-24

**Authors:** Amad N Khan, Salema Khalid, Hussain I Khan, Mehnaz Jabeen

**Affiliations:** 1 Aga Khan University Medical College, Karachi, Pakistan; 2 Aga Khan University Hospital, Research Office, Karachi, Pakistan

## Abstract

**Background:**

Living in a world greatly controlled by mass media makes it impossible to escape its pervading influence. As media in Pakistan has been free in the true sense of the word for only a few years, its impact on individuals is yet to be assessed. Our study aims to be the first to look at the effect media has on the body image of university students in a conservative, developing country like Pakistan. Also, we introduced the novel concept of body image dissatisfaction as being both negative and positive.

**Methods:**

A cross-sectional study was conducted among 7 private universities over a period of two weeks in the city of Karachi, Pakistan's largest and most populous city. Convenience sampling was used to select both male and female undergraduate students aged between 18 and 25 and a sample size of 783 was calculated.

**Results:**

Of the 784 final respondents, 376 (48%) were males and 408 (52%) females. The mean age of males was 20.77 (+/- 1.85) years and females was 20.38 (+/- 1.63) years. Out of these, 358 (45.6%) respondents had a positive BID (body image dissatisfaction) score while 426 (54.4%) had a negative BID score. Of the respondents who had positive BID scores, 93 (24.7%) were male and 265 (65.0%) were female. Of the respondents with a negative BID score, 283 (75.3%) were male and 143 (35.0%) were female. The results for BID vs. media exposure were similar in both high and low peer pressure groups. Low media exposure meant positive BID scores and vice versa in both groups (p < 0.0001) showing a statistically significant association between high media exposure and negative body image dissatisfaction. Finally, we looked at the association between gender and image dissatisfaction. Again a statistically significant association was found between positive body image dissatisfaction and female gender and negative body image dissatisfaction and male gender (p < 0.0001).

**Conclusions:**

Our study confirmed the tendency of the media to have an overall negative effect on individuals' body image. A striking feature of our study, however, was the finding that negative body image dissatisfaction was found to be more prevalent in males as compared to females. Likewise, positive BID scores were more prevalent amongst females.

## Background

Body image may be defined in simple terms as the way a person perceives or thinks about his body and how it appears to others [1]. Dissatisfaction with this is known as "body image dissatisfaction", a term that has been used in many articles on public health and psychology [1-3]. There is increasing pressure during adolescence for males and females to desire a body shape that conforms to the "ideal", i.e. a thin shape for women and a lean, muscular shape for men [2]. These perceived ideal body shapes are reinforced by the mass media and popular cultural icons. Internalization of body ideals that are perpetuated by the media can be a strong influence on body dissatisfaction, especially among females [3].

Body image perception varies greatly among males and females within the same age group [4]. In recent years, several studies have concluded that females have a higher tendency to adjudge themselves as not conforming to weight ideals as compared to men. This has been seen to be most prevalent in young adult females [5]. In addition to actual weight, perceived weight status is an important determinant of eating and weight-loss behaviour [6-10]. However, it is important to note that perceived weight does not always reflect actual weight status based on body mass index (BMI). Studies have shown that despite low rates of obesity, many university students, especially women, perceive themselves as overweight [8,11-14]. This is of concern, because inappropriate weight perceptions can lead to unhealthy behaviours including eating disorders [8,15-18]. Universities and colleges, on the other hand, represent an opportunity for reaching a large number of students to promote appropriate weight perceptions and healthy eating behaviours [16]. Several studies investigating the effect of exposure to the muscular male body ideal on body-focused self-perception among males have shown it to be associated with body dissatisfaction and muscularity dissatisfaction, especially in men with pre-existing muscularity concerns [19,20].

Although many articles study the effects of mass media on body image perceptions and dissatisfaction, mostly in females, very few have investigated the extent of the effect of mass media on body image perceptions specifically in university students, particularly in a developing country like Pakistan. Therefore, this study is necessary since media has been free in the true sense for only a few years and its impact is yet to be assessed. We conducted a systematic review and found no studies pertaining to body image in Pakistan. Moreover, all studies till date have assumed that body image dissatisfaction is always negative and leads to unhealthy eating practices and eating disorders. None have actually considered that body image dissatisfaction may lead to obese or underweight people trying to bring their BMI into the normal, healthy range and actually be a positive incentive. We realize that this dissatisfaction can lead to unrealistic expectations and unhealthy eating habits, but we have made provisions for appropriate reporting of this in the results section. We have proposed the new concept of positive and negative body image dissatisfaction and have aimed to see whether there is any association between either and media exposure, while controlling for peer pressure, a major confounder.

Our second objective was to see if there was any association between body image dissatisfaction and gender. If an association between media exposure and negative body image perception was apparent, we aimed to encourage the media to present more diverse and realistic images of people with positive messages about health and self-esteem. Although this may not eliminate eating disorders and unhealthy practices entirely, it would help reduce the pressure young adults feel to conform their bodies to one preconceived ideal.

## Methods

### Study Setting and Design

A cross-sectional study was conducted in 7 private universities (listed below) over two weeks in Karachi, Pakistan's largest and most populous city.

• Baqai Medical University

• Institute of Business Administration

• Aga Khan University

• College of Business Management

• Indus Valley School of Art and Architecture

• Institute of Business & Technology (BIZTEK)

• Iqra University

Ethical approval for the study was provided by the Aga Khan University Ethical Review Committee (ERC) and each participant gave written informed consent. Convenience sampling was used to select undergraduate students of both genders aged from 18 to 25. People who refused consent, had known psychiatric illnesses, metabolic disorders, endocrinological abnormalities, conditions impairing digestion, chronic infective conditions or malignancies were excluded from the study. The reason for selecting university students was to eliminate confounding bias of the level of education in the sample population. It was assumed that if all the members had a similar educational level it would make the effect of media on them comparable. Furthermore, body image perceptions haven't been looked at extensively in university students let alone in the setting of a developing country. Similarly, private universities were chosen to control for the socio-economic background of students.

### Operational Definitions and Study Tool

For our study, three operational variables were used. *Body image *was defined as an individual's own perception of their outlook; only physical aspects of body-image were considered, not the psychological aspects such as self-esteem, confidence, and personality disorders. *Media *included three categories: print media, television and internet; with each being further sub-divided into information, entertainment and sports. *Peer pressure *consisted of questions assessing how often, if at all, respondents felt they were affected by the attitudes of family and friends towards the respondents' body image.

A self-response questionnaire was used as the study tool. The questionnaire, designed in English, took between 3-5 minutes to fill. It was in English because English is an official language in Pakistan and the medium of instruction in the universities included in the study. It comprised of three sections; the first dealt with demographics, the second with media exposure and the third with body image perception and peer pressure. In the first segment, the individuals gave their basic demographics, their height and weight were measured and body mass index (BMI) calculated. In the second part, they were answered questions pertaining to the amount of media exposure they had from the print, TV and internet sources. The third component asked questions related to their current and ideal body image and the extent to which peer pressure affected their body image. Finally, questions based on the exclusion criteria were asked. A full copy of the questionnaire is available as "Additional File 1" for this paper.

### Sample size

A sample size of 783 was calculated using the formula for hypothesis testing for a two-sided population proportion at a level of significance of 5%.

## Results

A total of 950 questionnaires were distributed and 870 students (91.6%) responded. 784 students qualified for analysis after applying the exclusion criteria. Of these, 376 (48%) were males and 408 (52%) females. The mean age of males was 20.77 (+/- 1.85) years and females was 20.38 (+/- 1.63) years.

Of the 7 participating universities, two were medical and five were non-medical. About 100 male and female undergraduate students who satisfied the selection criteria and were available at the universities from 1^st ^March 2010 to 15^th ^March 2010 participated from each university. Our sample included 52% females and 48% males. These balanced percentages enabled us to compare the extent of media exposure in both genders.

**Figure 1:**
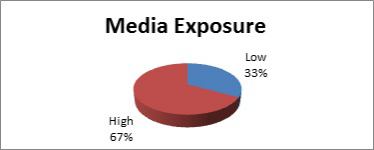
**Distribution of the sample on basis of media exposure**.

A scoring system was developed to measure the time spent on different modalities in each type of media. Media exposure cut-off score was kept 7 because a score of 7 is approximately equivalent to 21 hours or more per week of media exposure. The degree of media exposure was categorized into high exposure (>7) and low exposure (< 7). Consequently the scores were combined to give the overall exposure of an individual to mass media; the minimum score was arbitrarily set to 0 and the maximum score to 33. A full break-up of the media exposure in both males and females is presented in table 1, while figure 1 represents the entire sample with regards to media exposure for both genders combined. Interestingly, table 1 shows that males had higher exposure to the media than females (p = 0.001). The questionnaire and scoring system was piloted and validated amongst 100 students at the Aga Khan University Medical College.

**Table 1:** Media exposure vs. gender

	Low media exposure	High media exposure	**Chi-square**^**§**^
**Males **(n = 376)	101 (26.9%)^+^	275 (73.1%)	Chi-square = 11.9
	
**Females **(n = 408)	158 (38.7%)	250 (61.3%)	**p = 0.001**

+Percentages were calculated out of the total of males and females respectively.

§ Yates corrected values were used for better accuracy.

### Image dissatisfaction

**Figure 2:**
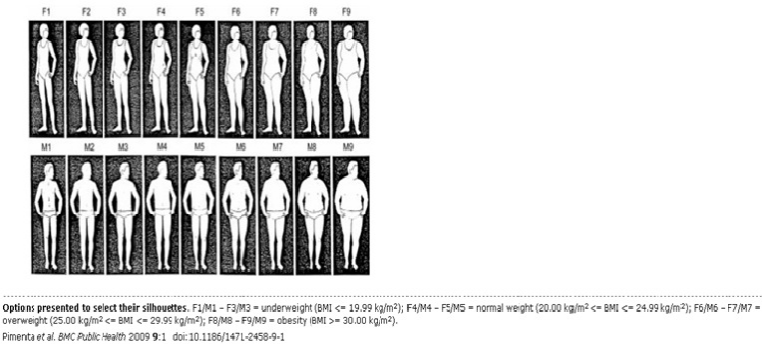
**Images shown to assess individuals' body image**.

In the second part of the questionnaire, the participants were given a set of nine drawings of increasing body size (Figure 2). These body depictions have been previously used in several studies to assess body image dissatisfaction [19,21-33]. Assessment of the body image diagrams was done by calculating the difference between current image and ideal image (C. Image - I. Image).

Based on our results we found out that the ideal body figure for both males and females was 4 (52.4% and 54.5% respectively). The difference between the current and ideal images was the "body image dissatisfaction (BID) score". This gave us a measure of the level of dissatisfaction with body image in individuals - the greater the scores, the greater the dissatisfaction. BID scores were then evaluated as positive or negative by keeping in mind the respondents' BMI, calculated from their measured height and weight. Their BID score was considered positive if individuals who had a BMI of a) greater than 25 wanted to lose weight (had a positive C-I and an ideal that was either image 4 or 5); b) less than 20 and wanted to gain weight (had a negative C-I and an ideal image of 4 or 5) and c) 20-25 who were satisfied with their body image (had a C-I of 0). Similarly, a BID score was considered negative if individuals had a BMI of a) less than 20 and wanted to stay the same or lose further weight (had a C-I of 0 or negative); b) 20-25 and wanted to lose weight (had a positive C-I) and c) more than 25 and wanted to stay the same or gain more weight (had a C-I of 0 or negative). Also included in the negative category were people who were overweight and wanted to lose too much weight (had an ideal image less than 4) as well as those who were underweight and wanted to gain too much weight (had an ideal image greater than 5). We realize that those individuals who had a BID score of zero were not dissatisfied with their body image but we feel that depending on their actual BMI it was fair to classify them as having a positive or negative BID because individuals' weights fluctuate about a certain level (just like any physiological homeostatic process) and a certain degree of dissatisfaction whether positive or negative keeps them in check.

Out of the 784 eligible students, 358(45.6%) had a positive BID score while 426(54.4%) respondents had a negative BID score. Out of those who had positive BID scores, 93(26.0%) were male and 265(74.0%) were female. Out of those with a negative BID score, 283(66.4%) were male and 143(33.6%) were female.

### How much does your family's opinion of you influence your self-perceived body image?

Out of the total of the 408 females, 224(54.9%) answered that their family's opinion doesn't influence their body image at all, 86(21.1%) were influenced slightly, 60(14.7%) were moderately influenced and only 38(9.3%) were greatly influenced.

From the total of the 376 males, 155(41.2%) answered that their family's opinion doesn't influence their body image at all. 102(27.1%) said that it influenced them slightly, 69(18.4%) were moderately influenced, and only 50(13.3%) said that it greatly influences them.

### How much does your friend's/colleague's opinion of you influence your self-perceived body image?

Out of the total of the 408 females, 220(53.9%) answered that their friend's/colleague's opinion doesn't influence their body image at all. 90(22.1%) said that it influenced them slightly, 56(13.7%) said that it moderately influences them and 42(10.3%) said that it greatly influences them.

From amongst the total of the 376 males, 145(38.6%) answered that their friend's/colleague's opinion doesn't influence their body image at all. 122(32.4%) said that it influenced them slightly, while 62(16.5%) said that it moderately influences them and 47(12.5%) said that it greatly influences them.

For both of these questions, the answers given by the participants led to the calculation of a "peer pressure score (PPS)". For those who answered "not at all", a PPS of 1 was given. "Slightly influenced" was given a score of 2, "Moderately influenced" a score of 3 and "Greatly influenced" a score of 4. The PPS was calculated by adding the scores of both the questions. Low peer pressure score was 4 and below, and high PPS was 5 and above. The break-up of PPS according to gender is given in table 2, which shows that women were more affected by peer pressure than were males (p < 0.0001).

**Table 2:** Peer pressure by gender

	Low PPS	High PPS	**Chi-square**^**§**^
**Males **(n = 376)	310 (82.4%)^+^	66 (17.6%)	Chi-square = 36.0
	
**Females **(n = 408)	257 (63.0%)	151 (37.0%)	**p < 0.0001**

*PPS = Peer pressure score

+Percentages were calculated out of the total of males and females respectively.

§ Yates corrected values were used for better accuracy.

Then, in order to eliminate the major confounding factor of peer pressure's effect on body image, we looked at the association between media exposure and BID scores in both the high PPS and low PPS groups. The level of significance for all p-values was kept at <0.01. As the results were similar in both groups, i.e. low media exposure meant positive BID scores and vice versa in both groups, this showed a positive association between high media exposure and negative body image dissatisfaction. Table 3 shows these results.

**Table 3:** Media exposure in both high and low PPS groups vs. BID Scores.

	Positive BID* **Scores (n = 358)**	Negative BID Scores (n = 426)	**Chi Square Values**^**§**^
Low Media Exposure	190 (53.0%)^+^	68 (16.0%)	Chi-square = 119.7
	
High Media Exposure	168 (47.0%)	358 (84.0%)	**p < 0.0001**

**In Low PPS**^**Group (n = 567)**			

Low Media Exposure	134 (37.4%)	52 (12.2%)	Chi-square = 77.7
	
High Media Exposure	123 (34.3%)	257 (60.3%)	**p < 0.0001**

			

**In High PPS Group (n = 217)**			

Low Media Exposure	54 (15.1%)	18 (4.2%)	Chi-square = 33.84
	
High Media Exposure	47 (13.1%)	99 (23.2%)	**p < 0.0001**

^PPS = Peer pressure score

*BID = Body Image Dissatisfaction Score.

§Chi square test was applied here to find significant results showing that high media exposure correlates with negative BID scores. Yates corrected values were used for better accuracy.

+The percentages are calculated by dividing the numbers by the totals of the BID groups

Lastly, we looked at the association between gender and image dissatisfaction. We found a positive association between positive body image dissatisfaction in female gender and negative body image dissatisfaction in male gender (p < 0.0001). This is shown in table 4.

**Table 4:** Association of Image Dissatisfaction with Gender

	Positive BID* Score	Negative BID Score	**Chi Square Values **^**§**^
**Males**	93 (24.7%)^^^	283 (75.3%)	Chi-square = 125.94
	
**Females**	265 (65.0%)	143 (35.0%)	**p < 0.0001**

*BID = Body Image Dissatisfaction Score.

§ Yates corrected values were used for better accuracy.

^Percentages were calculated out of the total of males and females.

## Discussion

### Cultural and religious background of the population studied

A 97% Muslim majority makes Pakistan the second-most populous Muslim country in the world. Pakistani society is largely hierarchical, with high regard for traditional Islamic values. Some urban families have grown into a nuclear family system because of the socio-economic constraints imposed by the traditional joint family system [34]. Recent decades have seen the emergence of a middle class in cities like Karachi with an average annual income of US$10,000 [35] that wish to move in a more centrist direction, as opposed to the north-western regions that remain highly conservative and dominated by centuries-old regional tribal customs. With regards to female fashion though, whether in the cities or the rural areas of Pakistan, most women dress in accordance with Islamic tradition - fully covered and with loose fitting clothes. It is important to note that though there are similarities between the Pakistani and Indian societies, Pakistan is much more conservative, as compared to the more liberal, secular India. As our study was set amongst seven private-sector universities in Karachi, our sample consisted of educated, middle-class, Muslim students in the backdrop of a conservative yet gradually progressing society of Karachi.

### Image dissatisfaction and Media exposure

Our study shows that individuals with a high media exposure had a higher statistically significant prevalence of negative body image dissatisfaction (84.0% of the negative BID group) compared to those with lower media exposure (16.0% of the negative BID group.

This result is corroborated by numerous studies conducted in the West. Results from a study conducted by the Department of Psychology, Kenyon College, Gambier, support the socio-cultural perspective that mass media promulgates a slender ideal that elicits body dissatisfaction[4]. Studies show that females are more likely to perceive a lean body as ideal, which may be fuelled by images of thin women portrayed in the media [36-39]. In a recent study among female college students in the United States, 39% of normal weight students named media as a source of pressure to be a certain weight [40]. This is of public health concern, because females who restrict their food intake in order to achieve or maintain a desired body weight may have inadequate intake for optimal health and may develop eating disorders [7]. In a study by Wardle and colleagues [11], about 50% of all female students from 22 countries were trying to lose weight, although only 5% had a BMI ≥ 25 kg/m^2^. As several studies have found high rates of unhealthy eating behaviours in university students, prevention programs for high risk female students may be appropriate [16].

A literature review on body image and eating disorders amongst Japanese adolescents showed a prevalence ranging from 0.025% to 0.2% for anorexia and from 1.9% to 2.9% for bulimia [41]. The authors felt that although eating disorders have been previously regarded as peculiar to Western society, they are now more of a global issue. They were of the opinion that since the aetiology of eating disorders is related to societal norms, culture and ethnicity, their study requires an understanding of body image disturbance in different cultural contexts. This aspect has been highlighted by our study and more studies which focus on the prevalence of eating disorders in addition to body image are needed.

Our study shows that in the setting of a developing country, media has a significant negative effect on body image dissatisfaction amongst university students. Prevention programs for such aforementioned students can be implemented to promote healthy body image perceptions and eating practices.

### Body image dissatisfaction and gender

With regards to the difference in image dissatisfaction in males and females, our study produced interesting results. In males, the prevalence of negative body image dissatisfaction was higher (283/376 = 75.3%) as compared to females (143/408 = 35.0%). On the contrary, women had a higher prevalence of positive BID compared to men - 265 (65.0%) vs. 93 (24.7%) respectively. This is in contrast to other literature on this topic which shows that body image dissatisfaction (assumed to be negative only) is more prevalent in women than men [42-45]. In fact, according to a study conducted in America, many contemporary American women covet an unrealistically thin body build for themselves, a phenomenon that could be detrimental to their emotional and physical health [5].

Table 5 summarises the findings of studies from different populations and compares them with our study. It shows that in all other populations, females have higher rates of body image dissatisfaction (assumed to be only negative). There is no single unified scale to quantify this parameter and hence the disparity in results could be attributed to this fact. However, we feel this is not the case because in essence all the scales are looking to quantify the same variable - body image dissatisfaction. The fact that all the studies based their findings on similar questions ascertaining the subjects' body image, combined with the highly significant results in all the papers, can only mean that the literature is correctly in agreement on body image dissatisfaction being higher in women in the West as well as Eastern but less conservative populations like India. In our setting, the disparity of men having higher negative body image dissatisfaction could be attributed to various socio-cultural factors e.g. covering of the full body and hair in loose fitting clothes by women. These result in a lower pressure on women to look like the ideal females presented by the media. With no similar cultural and religious restraints on males, they have an opportunity of achieving the ideal body image as portrayed by the media and consequently show greater dissatisfaction with their body image. Overindulgence in this dissatisfaction becomes negative in the majority of males. In a study conducted on Body Dysmorphic Disorder (BDD) in university students of Pakistan, the authors found that as the study population was in a conservative, Muslim country, females expressing concern over the size of legs/thighs and breasts was much less as compared to similar western studies [46]. The authors felt that females could be hesitant in reporting these foci of concern even if they were preoccupied within them, resulting in the discrepancy. Although these authors share our opinion, we realize further research is required to support this hypothesis with our study serving as a potential starting point.

**Table 5:** Body image dissatisfaction amongst different populations

Author	Population Details	Body Image Dissatisfaction (BID)
Wilkosz ME et al. [52]	Californian adolescents (n = 1807, 55.3% girls and 44.7% boys, aged 12-17)	24% of **girls **had BID compared with 22% of boys (p < .05)

Lobera IJ et al. [53]	Spanish university students (n = 417, 33.57% men and 66.43% women, mean age = 21.62)	Negative correlation with BIQLI-SP* solely in the case of **women **(r = -0.13; P, 0.05). BMI also negatively correlated with BIQLI-SP only in **women **(r = -0.20; P, 0.01)

Stigler MH et al. [54]	Indian 8^th ^and 10^th ^graders (n = 1818, 60% boys and 40% girls, mean ages = 13.9 years old and 15.8 years old for each grade respectively)	**Girls **were more likely than boys to perceive themselves as overweight (p = 0.047) and to have low body satisfaction (p = 0.052)

Khan AN et al. (present study)	Pakistani university students (n = 784, 48% males and 52% females, mean age = 20.6)	75.3% **males **as compared to only 35.0% females had negative BID, (p < 0.0001)

*(BIQLI-SP): Spanish version of the Body Image Quality of Life Inventory.

### Peer pressure

In a study conducted in Malaysia, family members, followed by friends, were found to be the main sources of advice concerning body shape problems [1]. Several other studies have also reported the important roles played by parents and peers in influencing changes in body image among adolescents [47-50] and we felt it was necessary to eliminate the major confounding bias of peer pressure so that we could solely look at the effect of media on body image.

From our results, we saw that females were more affected by peer pressure as compared to males (37% vs. 17.6%, p < 0.0001). -Our results were similar to a study conducted in Denver, USA, in which the authors found that late adolescent girls experienced greater interpersonal pressure to be thin than boys in relationships with mothers and peers [51]. The Malaysian study mentioned before also had results that concurred with ours [1]. Therefore, our results regarding peer pressure are corroborated by both Western and Eastern literature.

### Limitations

One limitation to our study was the validity of the responses of the respondents. We had no means of confirming either their media exposure or peer pressure. In the feedback that we got from the participants, they mentioned difficulty in estimating their media exposure in hours per week. Moreover, a major limitation in the use of the BMI is that it does not differentiate between muscle and fat mass [1].

## Conclusions

In concurrence with other studies, our study confirmed the tendency of the media to have an overall negative effect on peoples' body image. It also showed that a large proportion of individuals had high amounts of media exposure - more in men than in women. Our study has helped us to conclude that in a developing country like Pakistan, high exposure to media has a statistically significant negative effect on body image dissatisfaction of young university students.

A striking feature of our study was the finding that negative body image dissatisfaction was found to be more prevalent in males as compared to females. On the contrary, females had a higher prevalence of positive body image dissatisfaction. This is in contrast to the results of all the other studies that were reviewed on this topic. Therefore, further studies need to be carried out in other parts of Pakistan and similar conservative developing countries to shed more light on this issue.

## Competing interests

The authors declare that they have no competing interests.

## Authors' contributions

ANK and SK designed the questionnaire and distributed it amongst the participants.

HIK and MJ helped in the write-up and review of the article. All the authors read and approved the final manuscript.

## Pre-publication history

The pre-publication history for this paper can be accessed here:

http://www.biomedcentral.com/1471-2458/11/379/prepub

## Supplementary Material

Additional file 1 **Body Image Questionnaire**. Click here for file
