# Supplementary material for: Impact of today's media on university student's body image in Pakistan: a conservative, developing country's perspective
Source: BMC Public Health. 2011 May 24;11:379. doi: 10.1186/1471-2458-11-379 (PMC3119165; doi:10.1186/1471-2458-11-379)
Supplement: Additional file 1 — Body Image Questionnaire. [file 1471-2458-11-379-S1.DOC]

Questionnaire

1. Date:
2. Year in which the participant is enrolled:
3. Age on your last birthday:
4. How many brothers and sisters do you have? _________
5. Your position in the family:
   1. Youngest
   2. Middle
   3. Eldest
   4. Only Child

Nowadays, people tend to monitor their weight on a more regular basis than previously.

1. Weight: ____kg Height: ____m BMI: ______kg/m^2^

In today’s world, mass media has become an important part of our lives.

1. On television, *per week*, you spend this much time watching:

a) Informative programs <20 and more > <5-20 hours > <1-5 hours > < None >

b) Entertainment programs <20 and more > <5-20 hours > <1-5 hours > < None >

c) Sports <20 and more > <5-20 hours > <1-5 hours > < None >

d) Other (specify) _______________________________________

1. On the internet, *per week*, you spend this much time surfing:

a) Informative sites <20 and more > <5-20 hours > <1-5 hours > < None >

b) Entertainment sites <20 and more > <5-20 hours > <1-5 hours > < None >

c) Sports Sites <20 and more > <5-20 hours > <1-5 hours > < None >

d) Social Networking <20 and more > <5-20 hours > <1-5 hours > < None >

e) Other (specify) ­­­­­­­­­­­­­­­­­­­­­____________________________________________

1. How often do you read:

a) Sports magazines <20 and more > <5-20 hours > <1-5 hours > < None >

b) Entertainment magazines <20 and more > <5-20 hours > <1-5 hours > < None >

c) Informative magazines <20 and more > <5-20 hours > <1-5 hours > < None >

d) Newspapers <20 and more > <5-20 hours > <1-5 hours > < None >

e) Other (specify) ­­­­­­­­­­­­­­­­­­­­­____________________________________________

(For the following questions, use this scale.)

0=Not at all, 1=Slightly, 2=Moderately, 3=Greatly

1. How much does your family’s opinion of you influence your self-perceived body image? [0] [1] [2] [3]
2. How much does your friend’s/colleague’s opinion of you influence your self-perceived body image? [0] [1] [2] [3]
3. Out of these, which one is your ideal figure and which image describes your current state.

Current ____ Ideal: ____

1. Do you suffer from any of the following psychiatric illnesses?

- Depression
- Schizophrenia
- Brief reactive psychosis
- Dissociative disorder
- Bipolar disorder
- Any other (please specify) __________________________________________
- None

1. Do you suffer from any of the following conditions?

- Diabetes
- Thyroid abnormalities
- Tuberculosis
- Cancer
- Others (please specify) ­­­­­­­­­­­­­______­­­­­­­­­­­­­­­­­_______________________________________
- None

1. Does anyone in your family suffer from any chronic (long term) medical condition?

______________________________________________________________________________

1. Has anyone in your family had any surgical procedure done?

______________________________________________________________________________
